# Bidirectional association between asthma and migraines in adults: Two longitudinal follow-up studies

**DOI:** 10.1038/s41598-019-54972-8

**Published:** 2019-12-04

**Authors:** So Young Kim, Chanyang Min, Dong Jun Oh, Jae-Sung Lim, Hyo Geun Choi

**Affiliations:** 1 Department of Otorhinolaryngology-Head & Neck Surgery, CHA Bundang Medical Center, CHA University, Seongnam, Korea; 2 Hallym Data Science Laboratory, Hallym University College of Medicine, Anyang, Korea; 3 Graduate School of Public Health, Seoul National University, Seoul, Korea; 4 Department of Internal medicine, Asan Medical Center, University of Ulsan College of Medicine, Seoul, Korea; 5 Department of Neurology, Hallym University Sacred Heart Hospital, Anyang, Korea; 6 Department of Otorhinolaryngology-Head & Neck Surgery, Hallym University College of Medicine, Anyang, Korea

**Keywords:** Migraine, Asthma

## Abstract

The objective of this study was to evaluate the bidirectional association between asthma and migraines using control subjects matched by demographic factors. The Korean Health Insurance Review and Assessment Service - National Sample Cohort from 2002 to 2013 was used. In study I, 113,059 asthma participants were matched with 113,059 control I participants. In study II, 36,044 migraine participants were matched with 114,176 control II participants. The hazard ratios (HRs) of migraines in the asthma patients (study I) and asthma in the migraine patients (study II) were analyzed using stratified Cox proportional hazard models after adjusting for depression and the Charlson comorbidity index. In study I, 5.3% (6,017/ 113,059) of the asthma group and 3.4% (3,806/ 113,059) of the control I group had migraines (P < 0.001). The asthma group demonstrated an adjusted HR of 1.47 for migraine (95% confidence interval (CI) = 1.41–1.53, P < 0.001). In study II, 15.4% (5,548/36,044) of the migraine group and 10.6% (15,271/144,176) of the control group had asthma (P < 0.001). The migraine group showed an adjusted HR of 1.37 for asthma (95% CI = 1.33–1.41, P value < 0.001). Asthma and migraines are reciprocally associated.

## Introduction

Asthma is a chronic airway disease associated with clusters of respiratory symptoms, including wheezing and dyspnea. Asthma is a common disease with different prevalences according to ethnicity that range from approximately 7% to 18.0%^1^. The incidence of asthma in the adult population (>20 years old) was estimated to be approximately 3.63–6.07 per 1,000 people in Korea^2^. Because the diagnosis of asthma is based on the functional deterioration of the lower airway, the underlying pathophysiologic causes of asthma have been reported to vary among asthmatic patients^3^. According to the pathophysiologic mechanisms, asthma is classified as two major endotypes of T-helper type 2 cell (TH2)-high and TH2-low diseases^4^. Thus, in addition to TH2-high related diseases, such as allergic rhinitis, other immune-related diseases could complicate asthma. In addition, both genetic factors and environmental triggering factors have been suggested to be related to asthma^5,6^.

Migraine disorder is another chronic disease characterized by multiple symptoms of headache and/or aura. As many as approximately 1.04 billion people suffer from migraines worldwide (95% uncertainty interval = 1.00–1.09)^7^. In Korea, approximately 6.0% of the adult population (19–69 years) suffers from migraines^8^. Migraines are characterized by recurrent episodic symptom sequences of a premonitory phase, an aura phase, a headache phase, and a postdrome phase^9^. The environmental triggering factors can hyper-activate the hypothalamic-pituitary-adrenocortical axis and autonomic nervous system in migraine susceptible subjects^10^. These hormonal and nervous changes induce vasodilation, the release of vascular factors, including growth factors, cytokines, nitric oxide, norepinephrine and calcitonin gene-related peptide, and interaction with endothelial cells, resulting in migraine symptoms^11^. The pathophysiology of the hormonal imbalance and environmental triggering factors are common in both migraines and asthma^12–14^. Due the these shared pathophysiology and recurrent symptoms, asthma was hypothesized as a acephalgic migraine or pulmonary migraine^15,16^. In addition, several epidemiologic studies reported the high rate of asthma in migraine patients and vice versa^17,18^.

Clinically, a considerable portion of asthma patients have comorbid headaches^19,20^, and it was questionable which disease was the first event. We postulated that asthma and migraine have reciprocal relationships with each other. To prove this hypothesis, we designed two independent studies. In study I, asthma patients were evaluated for the risk of subsequent migraine and compared to a nonasthmatic control group. In contrast, study II was designed to investigate the subsequent occurrence of asthma in migraine patients compared to a nonmigraine control group. No previous study has concurrently evaluated the bidirectional association between asthma and migraine. In addition, the control group subjects were matched with the study group subjects by age, sex, income, and region of residence. We adjusted for depression and the Charlson comorbidity index (CCI) to exclude potential confounding effects.

## Results

### Study I

The time duration from the index date to a migraine was 43.8 months (SD = 33.5) in the asthma group and 43.0 months (SD = 34.5) in the control I group. The rate of migraine was higher in the asthma group (5.3% [6,017/113,059]) than in the control I group (3.4% [3,806/113,059], P < 0.001, Table 1). The rate of migraines with aura and migraines without aura in the asthma group were also higher than those in the control I group (S1 Table). The general characteristics (age, sex, income, and region of residence) of the participants were exactly the same due to matching (P = 1.000).

**Table 1:** General Characteristics of the Participants.

Characteristics	Study I			Study II		
	Asthma (n, %)	Control I (n, %)	P-value	Migraine (n, %)	Control II (n, %)	P-value
Age (years)			1.000			1.000
20–24	4,464 (3.9)	4,464 (3.9)		1,862 (5.2)	7,448 (5.2)	
25–29	7,142 (6.3)	7,142 (6.3)		2,459 (6.8)	9,836 (6.8)	
30–34	10,653 (9.4)	10,653 (9.4)		3,294 (9.1)	13,176 (9.1)	
35–39	10,723 (9.5)	10,723 (9.5)		3,801 (10.5)	15,204 (10.5)	
40–44	10,477 (9.6)	10,477 (9.6)		4,311 (12.0)	17,244 (12.0)	
45–49	10,907 (9.4)	10,907 (9.4)		4,629 (12.8)	18,516 (12.8)	
50–54	10,652 (9.5)	10,652 (9.5)		3,905 (10.8)	15,620 (10.8)	
55–59	10,214 (9.0)	10,214 (9.0)		2,971 (8.2)	11,884 (8.2)	
60–64	10,314 (9.1)	10,314 (9.1)		2,696 (7.5)	10,784 (7.5)	
65–69	10,247 (9.1)	10,247 (9.1)		2,467 (6.8)	9,868 (6.8)	
70–74	8,075 (7.1)	8,075 (7.1)		1,818 (5.0)	7,272 (5.0)	
75–79	5,122 (4.5)	5,122 (4.5)		1,113 (3.1)	4,452 (3.1)	
80–84	2,811 (2.5)	2,811 (2.5)		491 (1.4)	1,964 (1.4)	
85+	1,258 (1.1)	1,258 (1.1)		227 (0.6)	908 (0.6)	
Sex			1.000			1.000
Male	42,172 (37.3)	42,172 (37.3)		9,336 (25.9)	37,344 (25.9)	
Female	70,887 (62.7)	70,887 (62.7)		26,708 (74.1)	106,832 (74.1)	
Income		1.000			1.000	
1 (lowest)	18,702 (16.5)	18,702 (16.5)		6,082 (16.9)	24,328 (16.9)	
2	16,791 (14.9)	16,791 (14.9)		5,627 (15.6)	22,508 (15.6)	
3	20,207 (17.9)	20,207 (17.9)		6,589 (18.3)	26,356 (18.3)	
4	25,745 (22.8)	25,745 (22.8)		8,109 (22.5)	32,436 (22.5)	
5 (highest)	31,614 (28.0)	31,614 (28.0)		9,637 (26.7)	38,548 (26.7)	
Region of residence			1.000			1.000
Urban	51,947 (49.5)	51,947 (49.5)		15,554 (43.2)	62,216 (43.2)	
Rural	61,112 (54.1)	61,112 (54.1)		20,490 (56.8)	81,960 (56.8)	
Depression	13,047 (11.5)	9,404 (8.3)	<0.001^*^	6,587 (18.3)	12,661 (8.8)	<0.001^*^
CCI (score)^†^			<0.001^*^			<0.001^*^
0	40,492 (35.8)	53,529 (47.4)		12,614 (35.0)	69,552 (48.2)	
1	16,714 (14.8)	14,827 (13.1)		5,062 (14.0)	20,465 (14.2)	
≥2	55,853 (49.4)	44,703 (39.5)		18,368 (51.0)	54,159 (37.6)	
Asthma	113,059 (100.0)	0 (0.0)	<0.001^*^	5,548 (15.4)	15,271 (10.6)	<0.001^*^
Migraine	6,017 (5.3)	3,806 (3.4)	<0.001^*^	36,044 (100.0)	0 (0.0)	<0.001^*^

^*^Chi-square test. Significance at P < 0.05.

^†^Charlson Comorbidity Index was calculated without pulmonary disease.

**Figure 1:**
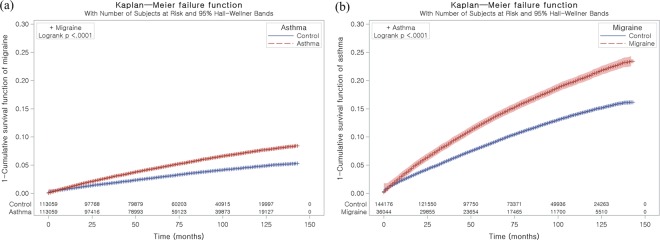
Kaplan-Meier survival analysis. (**a**) The group with asthma demonstrated a higher cumulative rate of migraines than the control I group. (**b**) The group with migraines demonstrated a higher cumulative rate of asthma than the control II group.

The adjusted HRs of migraines in the asthma group were 1.47 (95% CI = 1.41–1.53, P < 0.001, Table 2 and Fig. 1(a)). The adjusted HRs of migraines with aura and migraines without aura in the asthma group were 1.54 (95% CI = 1.33–1.78, P < 0.001) and 1.46 (1.40–1.53, P < 0.001), respectively. The adjusted HRs of migraine in asthma group were 1.96 (95% CI = 1.70–2.26) and 1.96 (95% CI = 1.63–2.37) after considering interactions with age and sex, respectively (S2 Table).

**Table 2:** Crude and adjusted hazard ratios (95% confidence interval) of asthma for migraines in study I.

Characteristics	Hazard ratios (HR)			
	Crude^†^	P-value	Adjusted^†^	P-value
HR for migraine				
Asthma	1.60 (1.53–1.66)	<0.001^*^	1.47 (1.41–1.53)	<0.001^*^
Control I	1.00		1.00	
HR for migraine with aura				
Asthma	1.68 (1.45–1.95)	<0.001^*^	1.54 (1.33–1.78)	<0.001^*^
Control I	1.00		1.00	
HR for migraine without aura				
Asthma	1.59 (1.52–1.65)	<0.001^*^	1.46 (1.40–1.53)	<0.001^*^
Control I	1.00		1.00	

^*^Cox-proportional hazard regression model. Significance at P < 0.05.

^†^Model stratified by age, sex, income, and region of residence.

^‡^Model adjusted for depression history and Charlson Comorbidity Index calculated without pulmonary disease.

In all subgroup analyses, higher adjusted HRs for migraine were observed in the asthma group (all P value < 0.001, Table 3). The adjusted HR was 2.05 (95% CI = 1.67–2.54) in <40-year-old men, 1.64 (95% CI = 1.49–1.79) in <40-year-old women, 1.73 (95% CI = 1.49–2.01) in 40–59-year-old men, 1.33 (95% CI = 1.24–1.43) in 40–59-year-old women, 1.45 (95% CI = 1.27–1.66) in ≥60-year-old men, and 1.39 (95% CI = 1.28–1.50) in ≥60-year-old women. These higher HRs of migraine in the asthma groups were also consistent during the follow-up periods (S3 Table).

**Table 3:** Subgroup analysis of the crude and adjusted hazard ratios (95% confidence interval) of asthma for migraines according to age and sex in study I.

Characteristics	Hazard ratios (HR) for migraine			
	Crude^†^	P-value	Adjusted^†‡^	P-value
Age <40 years, men (n = 22,102)				
Asthma	2.24 (1.82–2.76)	<0.001^*^	2.05 (1.67–2.54)	<0.001^*^
Control I	1.00		1.00	
Age <40 years, women (n = 43,862)				
Asthma	1.79 (1.63–1.96)	<0.001^*^	1.64 (1.49–1.79)	<0.001^*^
Control I	1.00		1.00	
Age 40–59 years, men (n = 31,696)				
Asthma	1.90 (1.63–2.21)	<0.001^*^	1.73 (1.49–2.01)	<0.001^*^
Control I	1.00		1.00	
Age 40–59 years, women (n = 52,804)				
Asthma	1.44 (1.35–1.55)	<0.001^*^	1.33 (1.24–1.43)	<0.001^*^
Control I	1.00		1.00	
Age ≥60 years, men (n = 30,546)				
Asthma	1.58 (1.38–1.80)	<0.001^*^	1.45 (1.27–1.66)	<0.001^*^
Control I	1.00		1.00	
Age ≥60 years, women (n = 45,108)				
Asthma	1.51 (1.39–1.63)	<0.001^*^	1.39 (1.28–1.50)	<0.001^*^
Control I	1.00		1.00	

^*^Cox-proportional hazard regression model. Significance at P < 0.05.

^†^Model stratified by age, sex, income, and region of residence.

^‡^Model adjusted for depression history and Charlson Comorbidity Index calculated without pulmonary disease.

### Study II

The time duration from the index date to asthma was 42.4 months (SD = 33.2) in the migraine group and 43.0 months (SD = 33.8) in the control II group. The rate of asthma was higher in the migraine group (15.4% [5,548/36,044]) than in the control II group (10.6% [15,271/144,176], P < 0.001, Table 1). The rate of asthma in the migraines with aura group and migraines without aura group was also higher than that in the control II group (S4 Table). The general characteristics (age, sex, income, and region of residence) of the participants were exactly the same due to matching (P = 1.000).

The adjusted HR of asthma in the migraine group was 1.37 (95% CI = 1.33–1.41, P value < 0.001, Table 4 and Fig. 1(b)). The adjusted HRs of asthma in the migraines with aura and migraines without aura groups were 1.39 (95% CI = 1.25–1.55, P < 0.001) and 1.37 (1.32–1.41, P < 0.001), respectively. The adjusted HRs of asthma in migraine group were 1.68 (95% CI = 1.49–1.88) and 1.80 (95% CI = 1.56–2.08) after considering interactions with age and sex, respectively (S2 Table). In all subgroup analyses, higher adjusted HRs for asthma were observed in the migraine group (all P value < 0.001, Table 5). The adjusted HR was 1.65 (95% CI = 1.40–1.95) in <40-year-old men, 1.42 (95% CI = 1.32–1.52) in < 40-year-old women, 1.57 (95% CI = 1.41–1.75) in 40–59-year-old men, 1.31 (95% CI = 1.25–1.38) in 40–59-year-old women, 1.45 (95% CI = 1.30–1.62) in ≥60-year-old men, and 1.27 (95% CI = 1.19–1.35) in ≥60-year-old women. These higher HRs for migraine in the asthma groups were also consistent during the follow-up periods (S5 Table).

**Table 4:** Crude and adjusted hazard ratios (95% confidence interval) of migraines for asthma in study II.

Characteristics	Hazard ratios (HR)			
	Crude^†^	P-value	Adjusted^†‡^	P-value
HRs for asthma in Migraine with/without aura (n = 180,220)				
Migraine	1.50 (1.45–1.54)	<0.001^*^	1.37 (1.33–1.41)	<0.001^*^
Control II	1.00		1.00	
HRs for asthma in Migraine with aura (n = 15,030)				
Migraine with aura	1.56 (1.40–1.73)	<0.001^*^	1.39 (1.25–1.55)	<0.001^*^
Control II	1.00		1.00	
HRs for asthma in Migraine without aura (n = 165,190)				
Migraine without aura	1.49 (1.45–1.54)	<0.001^*^	1.37 (1.32–1.41)	<0.001^*^
Control II	1.00		1.00	

^*^Cox-proportional hazard regression model. Significance at P < 0.05.

^†^Model stratified by age, sex, income, and region of residence.

^‡^Model adjusted for depression history and Charlson Comorbidity Index calculated without pulmonary disease.

**Table 5:** Subgroup analysis of the crude and adjusted hazard ratios (95% confidence interval) of migraines for asthma according to age and sex in study II.

Characteristics	Hazard ratios (HR) for asthma			
	Crude^†^	P-value	Adjusted^†‡^	P-value
Age <0 years, men (n = 14,485)				
Migraine	1.84 (1.56–2.16)	<0.001^*^	1.65 (1.40–1.95)	<0.001^*^
Control II	1.00		1.00	
Age <40 years, women (n = 42,595)				
Migraine	1.57 (1.46–1.68)	<0.001^*^	1.42 (1.32–1.52)	<0.001^*^
Control II	1.00		1.00	
Age 40–59 years, men (n = 20,030)				
Migraine	1.76 (1.57–1.96)	<0.001^*^	1.57 (1.41–1.75)	<0.001^*^
Control II	1.00		1.00	
Age 40–59 years, women (n = 59,050)				
Migraine	1.45 (1.38–1.53)	<0.001^*^	1.31 (1.25–1.38)	<0.001^*^
Control II	1.00		1.00	
Age ≥60 years, men (n = 12,165)				
Migraine	1.57 (1.41–1.75)	<0.001^*^	1.45 (1.30–1.62)	<0.001^*^
Control II	1.00		1.00	
Age ≥60 years, women (n = 31,895)				
Migraine	1.38 (1.39–1.47)	<0.001^*^	1.27 (1.19–1.35)	<0.001^*^
Control II	1.00		1.00	

^*^Cox-proportional hazard regression model. Significance at P < 0.05

^†^Model stratified by age, sex, income, and region of residence.

^‡^Model adjusted for depression history and Charlson Comorbidity Index calculated without pulmonary disease.

## Discussion

The present study demonstrated a bidirectional relationship between asthma and migraine in an adult population. The migraine patients demonstrated a 1.47 times higher hazard ratio of asthma than the control participants without migraines. However, the asthma patients showed a 1.37 times higher risk of migraines than the non-asthmatic control participants. All age and sex subgroups showed consistent results for both the risk of asthma in migraine patients and the risk of migraine in asthmatic patients. This study is the first to investigate the bidirectional relationship between asthma and migraines using matched control subjects and adjustment for the CCI. In addition, the migraine groups were classified based on the presence of aura.

In line with the present results, several previous studies have reported an association between asthma and migraine. A recent meta-analysis reported that asthmatic patients had 1.62 times higher odds of migraines than control subjects (95% confidence interval [95% CI] = 1.43–1.82)^21^. However, the migraine patients showed a 1.56 times higher risk of asthma than the control subjects (95% CI = 1.51–1.60, P < 0.00001)^21^. Using national health insurance data, a Taiwanese group demonstrated a 1.37-fold increased risk of asthma in migraine patients (95% CI = 1.21–1.56)^17^. Another study reported a 1.45-fold higher risk of migraine in asthma patients (95% CI = 1.33–1.59)^18^. However, their control groups were not matched with their study groups for socioeconomic factors and past medical histories. The study groups demonstrated higher rates of low economic status and comorbidities including rhinitis, sinusitis, atopic dermatitis, and chronic obstructive pulmonary disease. Although we adjusted for these covariates, the confounding effects of these covariates could not be excluded. Moreover, the importance of a past medical history of cardiovascular disease was not considered in these studies. The present study improved these previous findings by using control subjects who were matched for their past medical histories and demographic factors. Although future studies are warranted to delineate the biological pathophysiological mechanisms, a few plausible causes can be postulated from a number of prior studies.

The common pathophysiologic factors of inflammation and immune dysfunction between asthma and migraine could contribute to the bidirectional association between asthma and migraine. For instance, atopy, parasympathetic hyperactivity, and elevated neuropeptide secretion contribute to both asthma and migraines^22^. Allergic responses triggered by allergens, such as specific grass, pollen, and even hypertonic saline, evoke airway hypersensitivity responses mediated by mast cells in asthmatic patients^23^. In migraines, mast cells in the dura, which are activated by allergens, are presumed to excite meningeal nociceptors and initiate the activation of nearby trigeminal afferents^24^. In addition, parasympathetic hyperactivity is another shared feature of both asthma and migraine. An increased cholinergic tone in non-respiratory systems, which manifests as anxiety, reflux, heartburn, and abdominal pain, precedes asthma symptoms and has been suggested to trigger bronchospasm in asthma^25^. Parasympathetic afferents have been suggested to release acetylcholine, vasoactive intestinal polypeptide, and pituitary adenylate cyclase-activating polypeptide, which directly activate trigeminal nerves or provoke the degranulation of mast cells at meningeal levels in migraine^26^.

The shared triggering and environmental factors between asthma and migraine could induce a subsequent disease. Environmental factors including air pollutants impact both asthma and migraine. Air pollutants, including particulate matter, nitrogen dioxide, ozone, and carbon monoxide, are associated with the occurrence or aggravation of asthma, most likely due to oxidative stress affecting the airways^27^. Similarly, particulate matter, nitrogen dioxide, ozone, and carbon monoxide concentrations are related to the rate of emergency department visits for migraines (odds ratio = 1.053, 95% CI = 1.022–1.085 for nitrogen dioxide)^28^. Moreover, several lifestyle factors including obesity and smoking have been speculated to induce both asthma and migraine. In obese patients, due to the increased proportion of adipose tissue, systemic inflammation, metabolic changes, such as insulin resistance, and immune disturbances accompanied by microbiome changes occur and influence the occurrence of asthma^29^. For migraine, a recent meta-analysis reported that obesity increased the risk of migraine (odds ratio = 1.27, 95% CI = 1.16–1.37, P < 0.001)^30^.

Emotional stress, which is related to asthma and migraines, can promote the occurrence of other diseases. Stress or psychological distress, such as depression, due to asthma could increase an individual’s susceptibility to migraines and vice versa. Asthma was associated with a 1.35 times greater risk of depression in a previous study (95% CI = 1.31–1.40)^31^. Depression and stressful events are important modifiable risk factors for migraines^32^.

According to age and sex, all subgroups demonstrated a reciprocal association between asthma and migraines in the present study. Although the prevalence of migraines is higher in women than in men, the impact of asthma on migraines and the impact of migraines on asthma might be consistent regardless of sex^33^. In addition, both migraines with aura and migraines without aura were associated with the increased risk of asthma and vice versa in this study. Migraines with aura are suggested to be more associated with a family history of migraines and elevated risk of myocardial infarction, atrial fibrillation and stroke than migraines without aura^34–36^. Thus, migraines with aura might be more associated with cardiovascular and cerebrovascular compromise and related inflammatory responses. Therefore, the current study demonstrated higher HRs for migraines with aura in asthma patients (study I) and for asthma in migraines with aura patients (study II) compared to those with migraines without aura. In the present study, the risk of migraine associated with asthma (study I) was higher in both men and women (Table 3). There might have sex difference for the risk of migraine in asthma patients due to the higher rate of related comorbidities of migraine, thereby more other contributing factors for migraine, besides of asthma, in women than men. It was reported that women had more frequent headache and migraine-related comorbidities than men^37,38^. On the other hands, the risk of asthma related with migraine (study II) was also higher in both men and women (Table 5). The risk of asthma in migraine patients could be different according to sex due to the effect of sex hormone on the development of asthma. Estrogen was suggested to increase which testosterone was suggested to decrease Th2-mediated airway inflammation in asthma^39^. Both the risk of migraine associated with asthma and the risk of asthma related with migraine were decreased with aging in this study. The increase of comorbidities in elderly could attenuate the impact of asthma or migraine on the risk of migraine or asthma in this aged population. In the present study, the reciprocal associations between asthma and migraine were consistent after considering the interactions with age and sex (S2 Table).

The present study used a large study population, which provided a large number of control subjects. Due to this large population, random selection could be performed, including matching the control group participants for age, sex, income, and region of residence. Matching for socioeconomic status was crucial in this study based on health insurance codes because medical accessibility might be considerably determined by socioeconomic status. Because all Koreans are legally registered and reimbursed their medical costs by the Korean NHIS, no missing participants were anticipated. For the classifications of asthma and migraine, both diseases were diagnosed by physicians twice or more, and medication prescriptions were checked for asthma. Moreover, migraines were classified according to the presence of aura. Approximately 8% of migraine patients had aura in this study. This figure was lower than that reported in a previous study involving a Han Chinese population^33^. These objective and multiple inclusion criteria enhanced the fidelity of the present study. However, largely due to the limits of information included in the NHIS database, a few limitations of this study should be considered when interpreting the present results. Because the current study based on the health claim data, there is a limitation on the sensitivity and specificity of the definition of asthma. Approximately 20–70% of asthma patients were estimated to be undiagnosed and untreated, while about 30–35% of physician-diagnosed asthma patients were suggested not to have current asthma in a review study^40^, Subclinical or mild asthma or migraine patients might have been missed due to the lack of clinical visits. The severity or subtypes of asthma and migraine and each patient’s compliance to treatment could not be assessed. For instance, “allergic” asthma and “non-allergic” asthma could not be differentiated in this study. Detailed medication histories could not be considered for both diseases. The chronic use of nonsteroidal anti-inflammatory drugs (NSAIDs) in migraine patients could influence the occurrence of NSAID-induced asthma. In addition, lifestyle factors including obesity, smoking, and alcohol consumption were not available.

In conclusion, migraines increase the risk of asthma. Asthma was also associated with an elevated risk of migraine.

## Materials and Methods

### Study population and data collection

This study was ratified by the Ethics Committee of Hallym University (2017-I102). All analyses were conducted according to the guidelines and regulations of the ethics committee of Hallym University. The written informed consent was excused by the institutional review board.

The Korean National Health Insurance Service-National Sample Cohort (NHIS-NSC) was used as described in the prior studied^41,42^.

### Participant selection

The participants who were diagnosed with asthma (ICD-10: J45) or status asthmaticus (J46) from 2002 through 2013 were enrolled in this study, among the 1,125,691 cases with 114,369,638 medical claim codes. Among them, the asthma group was defined by a physician-diagnosis more than 2 times and the asthma-related medication histories, including inhaled corticosteroids (ICSs), ICSs combined with long-acting β2-agonists (LABAs), oral leukotriene antagonists (LTRAs), short-acting β2-agonists (SABAs), systemic LABAs, xanthine derivatives, and systemic corticosteroids (n = 230,764), similarly to a previous study^43^.

Migraine was defined based on the ICD-10 code (G43). Migraines were classified as migraines with aura and migraines without aura (ICD-10: G431). The participants who were treated for migraine ≥2 times (n = 45,587) from 2002 through 2013 were included. Both asthma and migraine were selected if participants were diagnosed with these conditions ≥2 times. The initial diagnosis was clinically determined by a diagnostic work-up including laboratory and imaging studies, and the confirmative diagnosis was performed at the second clinical visit.

#### Study I

**Figure 2:**
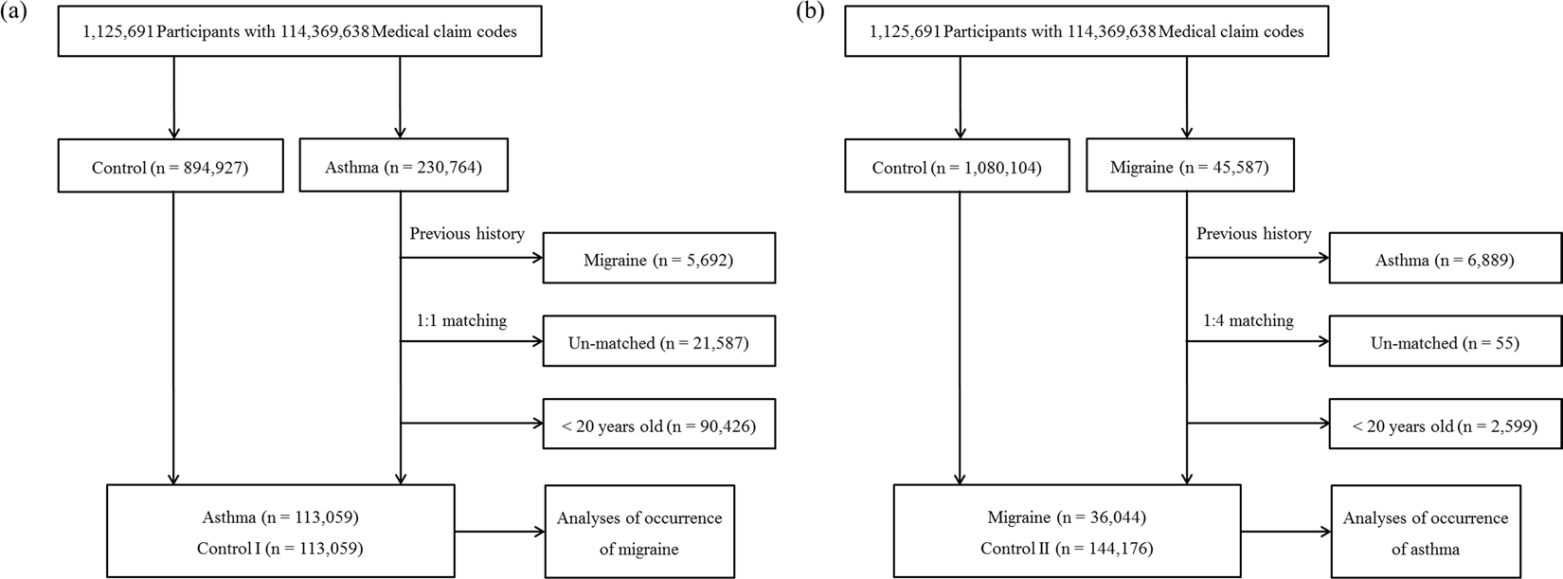
Schematic illustration of the participant selection process that was used in the present study. (**a**) Of a total of 1,125,691 participants, 113,059 asthma patients were matched with 113,059 control I participants by age group, sex, income group, region of residence, and past medical histories. (**b**) Of a total of 1,125,691 participants, 36,044 migraine patients were matched with 144,176 control II participants by age group, sex, income group, and region of residence.

Asthmatic participants were matched 1:1 with the participants (control I) without asthma. The control participants were randomly extracted from the original population as previous studies (n = 894,927)^41,42^. The participants were matched by age, group, sex, income group, region of residence, and past medical history (hypertension, diabetes, and dyslipidemia). In the asthma group, 5,692 participants were excluded. The asthma participants who lacked enough matched controls were excluded (n = 21,587). The <20 years of participants were excluded (n = 90,426). The mean follow up time was 80.7 months (Standard deviation [SD] = 41.1) for asthma group and 80.5 months (SD = 41.4) for control I group. As results, the 113,059 asthmatic participants and 113,059 control I subjects (nonasthmatic subjects) were included (Fig. 2(a)). The risks of migraine in the asthma and control I groups were analyzed.

#### Study II

Migraine patients were matched 1:4 with the control II group without migraine. The control participants were randomly extracted from the original population as previous studies (n = 894,927)^41,42^. The participants were matched for age, group, sex, income group, region of residence, and past medical history (hypertension, diabetes, and dyslipidemia). In the migraine group, 6,889 participants were excluded. The migraine participants who lacked enough matched controls were excluded (n = 55). The <20 years of participants were excluded (n = 2,599). The mean follow up time was 82.5 months (SD = 41.5) for migraine group and 81.9 months (SD = 41.7) for control II group. As results, the 36,044 migraine participants and 144,176 control II participants (nonmigraine participants) were included (Fig. 2(b)). The risks of asthma in migraine and control II groups were analyzed.

### Variables

The participants’ age group, income group, and region of residence was categorized as described in our prior studies^41,42^.

We adjusted for depression in the analysis due to its reciprocal association with asthma as revealed in our previous study^44^. Depression was classified as previously detailed^44^. CCI was counted, except for pulmonary disease, which includes asthma^45^.

### Statistical analyses

The general characteristics between the asthma and control groups (study I) and between the migraine and control groups (study II) were compared using the Chi-square test.

In study I, to analyze the hazard ratio (HR) of asthma for migraine, a stratified Cox proportional hazard model was used. In study II, to analyze the HR of migraine for asthma, another stratified Cox proportional hazard model was applied. In these analyses, crude and adjusted (depression and CCI) models were applied. These analyses were stratified for age, sex, income, and region of residence, and the 95% CIs were calculated. A Kaplan-Meier analysis and Log-rank test were conducted.

The participants were divided according age and sex (<40 years, 40–59 years, and 60+ years; male and female) to confirm these associations in different age and sex groups.

Two-tailed analyses were performed. The statistical significance was defined as P < 0.05. The SPSS v. 21.0 (IBM, Armonk, NY, USA) and SAS version 9.4 (SAS Institute Inc., Cary, NC, USA) were used.

## Supplementary information

Supplementary tables
