# Supplementary material for: Bidirectional association between asthma and migraines in adults: Two longitudinal follow-up studies
Source: Sci Rep. 2019 Dec 4;9:18343. doi: 10.1038/s41598-019-54972-8 (PMC6892888; doi:10.1038/s41598-019-54972-8)
Supplement: Supplementary file 1 — Supplementary tables [file 41598_2019_54972_MOESM1_ESM.docx]

**Bidirectional association between asthma and migraines in adults: Two longitudinal follow-up studies**

So Young Kim, MD^1^, Chanyang Min, PhD^2,3^, Dong Jun Oh, MD^4^, Jae-Sung Lim, MD^5^, Hyo Geun Choi, MD^2,6*^

^1^Department of Otorhinolaryngology-Head & Neck Surgery, CHA Bundang Medical Center, CHA University, Seongnam, Korea

^2^Hallym Data Science Laboratory, Hallym University College of Medicine, Anyang, Korea

^3^Graduate School of Public Health, Seoul National University, Seoul, Korea

^4^Department of Internal medicine, Asan Medical Center, University of Ulsan College of Medicine, Seoul

^5^Department of Neurology, Hallym University Sacred Heart Hospital, Anyang, Korea

^6^Department of Otorhinolaryngology-Head & Neck Surgery, Hallym University College of Medicine, Anyang, Korea

**Running title:** asthma and migraine

*Correspondence: [pupen@naver.com](mailto:pupen@naver.com)

**Key words:** Asthma; Migraine disorders; Risk factors; Cohort studies; Epidemiology

**S1** **Table** Distribution of migraines with/without aura in Study I.

| Characteristics | | Study I | | |
| --- | --- | --- | --- | --- |
|  |  | Asthma (n, %) | Control I (n, %) | P-value |
| Migraine with aura | | 485 (0.4) | 288 (0.3) | <0.001* |
| Migraine without aura | | 5,532 (4.9) | 3,518 (3.1) | <0.001* |

* Chi-square test, Significance at P < 0.05

**S2 Table** Crude and adjusted hazard ratios (95% confidence interval) of asthma for migraine in study I and of migraine for asthma in study II

| Characteristics | | | Hazard ratios | | | | | | | |
| --- | --- | --- | --- | --- | --- | --- | --- | --- | --- | --- |
|  |  | Crude† | | P-value | Model 1†‡ | P-value | Model 2§ | P-value | Model 3\|\| | P-value |
| **Study I** | | | | | | | | | | |
|  | Asthma | 1.60 (1.53-1.66) | | <0.001* | 1.47 (1.41-1.53) | <0.001* | 1.96 (1.70-2.26) | <0.001* | 1.96 (1.63-2.37) | <0.001* |
|  | Age*asthma |  | |  |  |  | 0.97 (0.96-0.99) | <0.001* |  |  |
|  | Sex*asthma |  | |  |  |  |  |  | 0.85 (0.77-0.94) | 0.002 |
|  | Age group |  | |  | 0.99 (0.99-1.00) | 0.115 | 1.01 (1.00-1.02) | 0.050* | 0.99 (0.99-1.00) | 0.116 |
|  | Sex (ref = women) |  | |  | 0.47 (0.45-0.50) | <0.001* | 0.47 (0.45-0.50) | <0.001* | 0.43 (0.39-0.46) | <0.001* |
|  | Income |  | |  | 0.98 (0.96-0.99) | 0.001* | 0.98 (0.96-0.99) | 0.001* | 0.98 (0.96-0.99) | 0.001* |
|  | Region of residence (ref = Rural) |  | |  | 0.90 (0.86-0.94) | <0.001* | 0.90 (0.86-0.94) | <0.001* | 0.90 (0.86-0.94) | <0.001* |
|  | Depression (ref = none) |  | |  | 1.84 (1.75-1.94) | <0.001* | 1.84 (1.75-1.94) | <0.001* | 1.84 (1.75-1.94) | <0.001* |
|  | CCI score |  | |  | 1.11 (1.10-1.12) | <0.001* | 1.11 (1.11-1.12) | <0.001* | 1.11 (1.10-1.12) | <0.001* |
|  | Control I | 1.00 | |  | 1.00 |  | 1.00 |  | 1.00 |  |
| **Study II** | | | | | | | | | | |
|  | Migraine | 1.50 (1.45-1.54) | | <0.001* | 1.37 (1.33-1.41) | <0.001* | 1.68 (1.49-1.88) | <0.001* | 1.80 (1.56-2.08) | <0.001* |
|  | Age* migraine |  | |  |  |  | 0.98 (0.97-0.99) | <0.001* |  |  |
|  | Sex* migraine |  | |  |  |  |  |  | 0.86 (0.79-0.93) | <0.001* |
|  | Age group |  | |  | 1.06 (1.06-1.07) | <0.001* | 1.07 (1.06-1.08) | <0.001* | 1.06 (1.06-1.07) | <0.001* |
|  | Sex (ref = women) |  | |  | 0.68 (0.65-0.70) | <0.001* | 0.68 (0.65-0.70) | <0.001* | 0.65 (0.62-0.68) | <0.001* |
|  | Income |  | |  | 0.98 (0.97-0.99) | <0.001* | 0.98 (0.97-0.99) | <0.001* | 0.98 (0.97-0.99) | <0.001* |
|  | Region of residence (ref = Rural) |  | |  | 0.97 (0.95-1.00) | 0.055 | 0.97 (0.95-1.00) | 0.055 | 0.97 (0.95-1.00) | 0.055 |
|  | Depression (ref = none) |  | |  | 1.25 (1.20-1.29) | <0.001* | 1.25 (1.20-1.30) | <0.001* | 1.25 (1.20-1.30) | <0.001* |
|  | CCI score |  | |  | 1.11 (1.10-1.11) | <0.001* | 1.11 (1.10-1.11) | <0.001* | 1.11 (1.10-1.11) | <0.001* |
|  | Control II | 1.00 | |  | 1.00 |  | 1.00 |  | 1.00 |  |

* Cox-proportional hazard regression model, Significance at P < 0.05

† The model was stratified for age, sex, income, and region of residence (except for covariates: age, sex, income, region of residence, depression history, and CCI score in model 1)

‡ Model 1 of asthma in study I and of migraine in study II was adjusted for depression history and CCI score. Other variables were adjusted for following covariates: In study I, model 1 was adjusted for age, sex, income, region of residence, depression history, and CCI score. In study II, model 1 was adjusted for age, sex, income, region of residence, depression history, and CCI score

§ In study I, model 2 was adjusted for age, age*asthma (interaction), sex, income, region of residence, depression history, and CCI score. In study II, model 2 was adjusted for age, age*migraine (interaction), sex, income, region of residence, depression history, and CCI score

|| In study I, model 3 was adjusted for age, sex, sex*asthma (interaction), income, region of residence, depression history, and CCI score. In study II, model 3 was adjusted for age, sex, sex*migraine (interaction), income, region of residence, depression history, and CCI score.

Abbreviation: CCI, Charlson comorbidity index

**S3 Table** Subgroup analysis of the crude and adjusted hazard ratios (95% confidence interval) of asthma for migraine according to the follow up periods in study I

| Characteristics | | Migraine | | | |
| --- | --- | --- | --- | --- | --- |
|  |  | Crude† | P-value | Adjusted†‡ | P-value |
| ≤ 1 year | | | | | |
|  | Asthma | 1.30 (1.19-1.42) | <0.001* | 1.21 (1.10-1.32) | <0.001* |
|  | Control I | 1.00 |  | 1.00 |  |
| 2 year | | | | | |
|  | Asthma | 1.85 (1.66-2.05) | <0.001* | 1.72 (1.54-1.91) | <0.001* |
|  | Control I | 1.00 |  | 1.00 |  |
| 3 year | | | | | |
|  | Asthma | 1.80 (1.60-2.02) | <0.001* | 1.65 (1.47-1.86) | <0.001* |
|  | Control I | 1.00 |  | 1.00 |  |
| > 3 year | | | | | |
|  | Asthma | 1.61 (1.52-1.70) | <0.001* | 1.48 (1.40-1.57) | <0.001* |
|  | Control I | 1.00 |  | 1.00 |  |

* Cox-proportional hazard regression model. Significance at P < 0.05

† Model stratified by age, sex, income, and region of residence

‡ Model adjusted for depression history and Charlson Comorbidity Index calculated without pulmonary disease.

**S4** **Table** Distribution of asthma in Study II.

| Characteristics | | Study II | |
| --- | --- | --- | --- |
|  |  | Asthma (n, %) | P-value |
| Migraine with aura (n = 15,030) | |  | <0.001* |
| Migraine with aura | | 485 (16.1) |  |
| Control II for migraine with aura | | 1,307 (10.9) |  |
| Migraine without aura (n = 165,190) | |  | <0.001* |
| Migraine without aura | | 5,063 (15.3) |  |
| Control II for migraine without aura | | 13,964 (10.6) |  |

* Chi-square test, Significance at P < 0.05

**S5 table** Subgroup analysis of crude and adjusted hazard ratios (95% confidence interval) of migraine for asthma according to follow up periods in study II

| Characteristics | | Asthma | | | |
| --- | --- | --- | --- | --- | --- |
|  |  | Crude† | P-value | Adjusted†‡ | P-value |
| ≤ 1 year | | | | | |
|  | Migraine | 1.37 (1.28-1.46) | <0.001* | 1.25 (1.17-1.34) | <0.001* |
|  | Control II | 1.00 |  | 1.00 |  |
| 2 year | | | | | |
|  | Migraine | 1.73 (1.60-1.87) | <0.001* | 1.59 (1.47-1.72) | <0.001* |
|  | Control II | 1.00 |  | 1.00 |  |
| 3 year | | | | | |
|  | Migraine | 1.51 (1.39-1.65) | <0.001* | 1.38 (1.26-1.50) | <0.001* |
|  | Control II | 1.00 |  | 1.00 |  |
| > 3 year | | | | | |
|  | Migraine | 1.49 (1.42-1.55) | <0.001* | 1.36 (1.30-1.42) | <0.001* |
|  | Control II | 1.00 |  | 1.00 |  |

* Cox-proportional hazard regression model, Significance at P < 0.05

† Stratified model for age, sex income, and region of residence

‡ Adjusted model for depression history and Charlson Comorbidity Index calculated without pulmonary disease.
